# DFT Calculations of Structure and IR Spectra of Gd_2_@C_82_ Endofullerenes

**DOI:** 10.3390/molecules31101756

**Published:** 2026-05-20

**Authors:** Igor V. Nechaev, Alexey V. Krisilov, Vladislav E. Chernov, Marina G. Holyavka

**Affiliations:** 1 Department of Chemistry, Voronezh State University, 394018 Voronezh, Russia; nechaev_iv@chem.vsu.ru; 2 Department of Physics, Voronezh State University, 394018 Voronezh, Russia; alexph@mail.ru; 3 Department of Biology and Medicine, Voronezh State University, 394018 Voronezh, Russia; holyavka@rambler.ru

**Keywords:** endohedral metallofullerenes, gadolinium, IR spectra

## Abstract

Dimetallofullerenes are obtained in synthesis in parallel to monometallofullerenes, but they are less studied because their yields are considerably lower. In this work, DFT modeling of Gd_2_@C_82_ endohedral metallofullerene (EMF) has been performed. A total of 32 isomers of Gd_2_@C_82_ have been established, the most stable of which form the C_2*v*_(9)-C_82_ structure. The similarity of the infrared (IR) spectra of the ground and excited states of C_2*v*_(9)-C_82_ is here established.

## 1. Introduction

Endohedral fullerenes are fullerenes with an atom or molecule placed inside the carbon cage, which screens the encapsulated particle from external electromagnetic and chemical interactions. Due to their unique properties, endohedral metallofullerenes (EMFs) have attracted considerable attention in fundamental science, having a wide range of possible applications. Studying endohedral metallofullerenes (EMFs) is of interest in the domain of nanotechnologies (e.g., single-molecule magnets [1,2,3] and single-molecule transistors [4]), alongside their biomedical [5,6] and other applications [7,8,9,10].

Endofullerenes were discovered in 1985, almost simultaneously with “empty” fullerenes [11]. Historically, La@C_60_ was the first to be synthesised. Until recently, lanthanide endofullerenes were the most abundant and available for study. The structure and IR spectra of C_60_ mono- and di-EMFs with (one or two) encapsulated lanthanide atoms have been calculated in several studies (see, e.g., [12,13,14]). Although La@C_82_ was the first EMF isolated in macroscopic quantities [11], the most thermodynamically stable metallofullerenes are formed by the C_82_ carbon cage. To date, endofullerenes of all lanthanides have been obtained, except promethium, which has no stable isotopes [15].

Endofullerene Gd@C_82_ has been the subject of considerable interest among researchers since its first synthesis. It is generally accepted that the most stable form of the Gd@C_82_ endofullerene is formed from the triplet C_82_ isomer with the C_2*v*_ symmetry group. However, there has been some debate for some time about the position of the Gd atom relative to the carbon cage, as discussed in Ref. [16]. Quantum chemical calculations employing pure density functionals (PBE, PW91, BLYP, BP86, and BPW91) indicate that the Gd atom resides along the C_2_ axis opposite the six-membered (hexagonal) ring [17]. In contrast, computations utilising the hybrid B3LYP functional reveal that this configuration constitutes a transition state characterised by an imaginary vibrational frequency. Furthermore, in the most stable form with C_*s*_ symmetry, the Gd atom is displaced closer to the C–C bond of the ring traversed by the C_2_ symmetry axis [18]. Ultimately, the controversy regarding the Gd atom’s location within the carbon cage was resolved both theoretically [19] and experimentally [20]. The authors of [19] performed detailed DFT calculations on the structures of Eu@C_82_ and Gd@C_82_, demonstrating that these metallofullerenes have a “normal” configuration similar to that observed in Sc@C_82_, Y@C_82_ and La@C_82_, in which the metal atom is located on the C_2_ axis. This configuration has also been confirmed experimentally [20] using XANES spectroscopy. According to Mizorogi and Nagase [19], the ground Gd@C_2*v*_(9)-C_82_ state has *M* = 7; the first excited state (*M* = 9) lies by ∼4 kJ/mol above.

Dimetallofullerenes are obtained in synthesis in parallel to monometallofullerenes, but their yields are considerably lower [21]. Di-EMFs M_2_@C_2*n*_ can sometimes correspond to carbide clusterfullerenes M_2_C_2_@C_2*n*−2_, and it is not possible to distinguish two classes of structures based on the mass-spectrometry data [22]. A number of M_2_@C_82_ EMFs were synthesised and isolated, such as Er_2_@C_82_ [23], Sc_2_@C82 [24], and Y_2_@C_82_ [25]. X-ray diffraction studies show that metal atoms are disordered among many positions along the belt of 10 continuous hexagons [26]. Also, there should be an M–M bond in these EMFs, and the formal charge of the cage is −4 [26].

DFT (density functional theory) calculations are widely used for modelling the endohedral fullerenes, both with a single atom [13] or a molecule [27] encapsulated inside the carbon cage. At the same time, the understanding of the electronic structure of C_82_ fullerenes is still far from complete [28]. In our previous works (see, e.g., [12,14,29] and references therein) we undertook a DFT analysis to compare the structures, symmetries, spins and dipole moments of lanthanide mono- and diendofullerenes by means of quantum chemical calculations and obtained their vibrational spectra in different spin states.

The aim of the present work is to investigate the structure and stability of Gd_2_@C_82_ isomers and to calculate the properties and IR spectra of several stable Gd_2_@C_82_ isomers. The calculation scheme is described in Section 3. The results of the calculations given in Section 2, where we present the geometric parameters of the calculated structures, list the most stable isomers and their thermodinamical properties. We present the vibrational spectra frequencies of the six most stable isomers and compare the IR spectra of the most stable Gd_2_@C_2*v*_(9)-C_82_ No. 1 isomer with those of empty C_2*v*_(9)-C_82_ fullerene. We also compare the IR spectra of the Gd_2_@C_2*v*_(9)-C_82_ No. 1 isomer in its ground (*M* = 15) and spin-excited (*M* = 17) states.

## 2. Results and Discussion

### 2.1. C_82_ Fullerene Isomers

**Figure 1:**
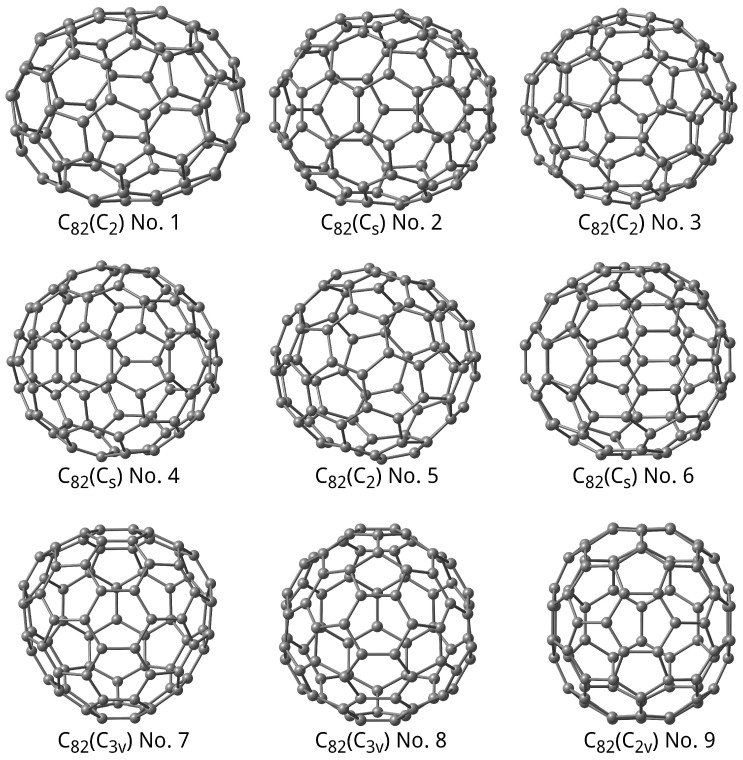
Optimised structure of C_82_ isomers.

Fullerene C_82_ has nine isomers fulfilling the isolated pentagon rule (IPR) [30,31]. Their optimised structure is shown in Figure 1, with designations given according to [30] [Table A.11, p. 256].

According to the calculations, the first six isomers have a singlet ground state and the last three have a triplet ground state. Table 1 summarises some properties of these isomers, including:

*M*, multiplicity of the ground state;
$G^{\circ }-G_{\min}^{\circ }$, difference in standard Gibbs energy between the current isomer and the isomer with the minimal $G^{\circ }$;
$E_{b}\left(C_{82}\right)$, binding energy per carbon atom;
$\Delta H_{f}^{\circ }\left(C_{82}\right)$, standard enthalpy of formation;
$\Delta G_{f}^{\circ }\left(C_{82}\right)$, standard Gibbs energy of formation;
IP, adiabatic ionisation potential;
EA, adiabatic electron affinity.

The values of $\Delta H_{f}^{\circ }$ and $\Delta G_{f}^{\circ }$ were estimated as

$$\begin{array}{ccc} \Delta H_{f}^{\circ }\left(C_{82}\right) & = & 82\Delta H_{f}^{\circ }\left(\mathrm{graphite}\right)+H^{\circ }\left(C_{82}\right)-82H^{\circ }\left(C\right);\\ \Delta G_{f}^{\circ }\left(C_{82}\right) & = & 82\Delta G_{f}^{\circ }\left(\mathrm{graphite}\right)+G^{\circ }\left(C_{82}\right)-82G^{\circ }\left(C\right), \end{array}$$

where $\Delta H_{f}^{\circ }\left(\mathrm{graphite}\right)$ and $\Delta G_{f}^{\circ }\left(\mathrm{graphite}\right)$ are the standard enthalpy and Gibbs energy of formation of graphite (717 kJ/mol and 671 kJ/mol, respectively [[32], pp. 5–9]).

**Table 1:** Calculated properties of C_82_ isomers.

No.	*M*	Symmetry Group	$G^{\circ }-G_{\min}^{\circ }$, kJ/mol	$E_{b}\left(C_{82}\right)$, kJ/mol	$\Delta H_{f}^{\circ }\left(C_{82}\right)$, kJ/mol	$\Delta G_{f}^{\circ }\left(C_{82}\right)$, kJ/mol	IP, eV	EA, eV
1	1	C_2_	31	701	1300	955	6.71	3.59
2	1	C_*s*_	26	701	1295	950	6.84	3.34
3	1	C_2_	0	702	1269	924	6.87	3.39
4	1	C_*s*_	10	701	1279	934	6.94	3.49
5	1	C_2_	24	701	1293	948	6.94	3.73
6	1	C_*s*_	36	701	1305	960	6.89	3.83
7	3	C_3*v*_	79	701	1349	1004	6.88	3.88
8	3	C_3*v*_	79	700	1354	1003	6.85	3.99
9	3	C_2*v*_	43	701	1314	967	6.86	3.95

The most stable isomer is structure No. 3 with C_2_ symmetry. The difference, $G^{\circ }-G_{\min}^{\circ }$, of Gibbs energy of the least and most stable isomer is 79 kJ/mol. No. 7–9 triplet structures are less stable than singlet structures. The obtained values of $E_{b}\left(C_{82}\right)$ indicate that all isomers of C_82_ are more stable than C_60_. Note that C_60_ has only one stable isomer C_60_-I_*h*_ (1) (with icosahedral symmetry). The rest of its isomers do not fulfil the isolated pentagon rule (IPR) and are much less stable [33]. Therefore, we present in Table 1 the physical properties of the C_82_ isomers and compare them with the properties of the above-mentioned C_60_-I_*h*_ (1) stable isomer, rather than perform additional internal intraseries comparisons among the C_60_ and C_82_ isomers.

The standard enthalpies of formation of C_82_ isomers lie in the range 1269–1354 kJ/mol and reflect the stability of these structures relative to graphite under standard conditions. The calculated values of $\Delta G_{f}^{\circ }\left(C_{82}\right)$ are less than the corresponding values of $\Delta H_{f}^{\circ }\left(C_{82}\right)$ by ∼350 kJ/mol, indicating an increase in entropy during the formation of C_82_ from graphite.

The ionisation potential (IP) and electron affinity energy (EA) determine the electron-donor and electron-acceptor properties of a compound. The high value of EA for C_60_ (2.81 eV) allows this fullerene to be actively used as an electron-acceptor in photovoltaics [26]. For the C_82_ isomers, the calculated EA values are higher than for C_60_ (Table 1), indicating even higher electron-acceptor properties of C_82_ as compared to those of C_60_. The IP values obtained for the C_82_ isomers are lower than those for C_60_ (7.57 eV).

### 2.2. Gd_2_@C_82_ EMF Isomers

**Figure 2:**
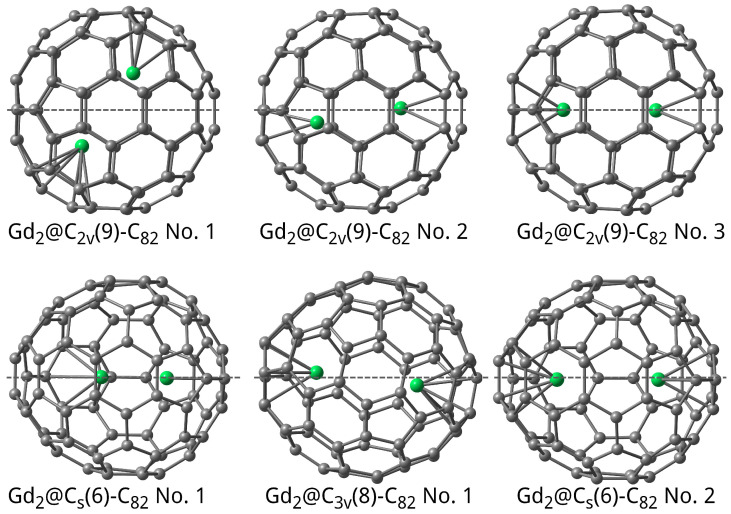
Optimised structure of the six most stable Gd_2_@C_82_ isomers. Dashed gray lines denote symmetry axes or C_82_ cage planes.

About 100 of the starting geometries were generated with different initial positions of Gd atoms relative to the C_82_ cage, followed by their further optimisation to determine possible isomers of Gd_2_@C_82_. The designation of the di-endofullerene isomers starts with the symmetry group of the parent “empty” C_82_ isomer followed by its serial number in parentheses (see, e.g., [34,35,36] and references therein). A total of 32 Gd_2_@C_82_ isomers were obtained, the 3 most stable of which correspond to the C_2*v*_(9)-C_82_ isomer. However, only the Gd_2_@C_2*v*_(9)-C_82_ No. 3 isomer (Figure 2) has a Gd atom on the C_2_ axis near the hexagonal ring as the most stable Gd@C_2*v*_(9)-C_82_ mono-EMF. The $G^{\circ }-G_{\min}^{\circ }$ difference between the most- and least-stable isomers is 159 kJ/mol. The multiplicity of the ground state for all isomers is 15.

Table 2 presents some properties of the six most stable isomers of Gd_2_@C_82_, including:

*M*, multiplicity of the ground state;
$G^{\circ }-G_{\min}^{\circ }$, the difference in standard Gibbs energy between the current isomer and the isomer with the minimal $G^{\circ }$ value;
$\Delta H_{e}^{\circ }$, standard enthalpy of encapsulation (inside the fullerene cage) per Gd atom;
$\Delta G_{e}^{\circ }$, standard Gibbs Energy of encapsulation (inside the fullerene cage) per Gd atom;
$r_{\mathrm{Gd}-\mathrm{Gd}}$, distance between gadolinium atoms;
$Q(C_{82})$, the charge of the C_82_ carbon cage calculated from Hirschfeld population analysis [37];
$S(C_{82})$, spin density of the C_82_ carbon cage;
IP, adiabatic ionisation potential;
EA, adiabatic electron affinity.

The calculated enthalpies and Gibbs energies of encapsulation (Table 2) indicate the formation of a strong bond between the Gd atom and the inner surface of the fullerene. It should be noted that the strongest bond between the Gd atom and the cage (556 kJ/mol per atom) is observed not for the most stable isomer, Gd_2_@C_2*v*_(9)-C_82_ No. 1, but for the structure Gd@C_3*v*_(8)-C_82_ No. 1, which is formed by the least stable isomer C_3*v*_(8)-C_82_. The difference between $\Delta G^{\circ }e$ and $\Delta H_{e}^{\circ }$ is ∼35–50 kJ/mol, indicating a decrease in entropy upon encapsulation of the Gd atom inside the C_82_ cage.

**Table 2:** Calculated properties of the six most stable Gd_2_@C_82_ isomers.

Isomer	*M*	$G^{\circ }-G_{\min}^{\circ }$, kJ/mol	$\Delta H_{e}^{\circ }$, kJ/mol	$\Delta G_{e}^{\circ }$, kJ/mol	$r_{\mathrm{Gd}-\mathrm{Gd}}$, pm	Q(Gd), a. u.	$S(C_{82})$	IP, eV	EA, eV
Gd_2_@C_2*v*_(9)-C_82_ No. 1	15	0	−542	−498	395.6	−4.527	−0.626	6.51	3.38
Gd_2_@C_2*v*_(9)-C_82_ No. 2	15	5	−539	−495	398.2	−4.509	−0.654	6.57	3.43
Gd_2_@C_2*v*_(9)-C_82_ No. 3	15	5	−539	−495	392.3	−4.510	−0.685	6.58	3.42
Gd_2_@C_*s*_(6)-C_82_ No. 1	15	7	−535	−490	377.1	−4.508	−0.634	6.62	3.06
Gd_2_@C_3*v*_(8)-C_82_ No. 1	15	10	−556	−510	417.2	−4.539	−0.566	6.63	3.20
Gd_2_@C_*s*_(6)-C_82_ No. 2	15	13	−531	−488	404.3	−4.548	−0.563	6.56	3.39

**Figure 3:**
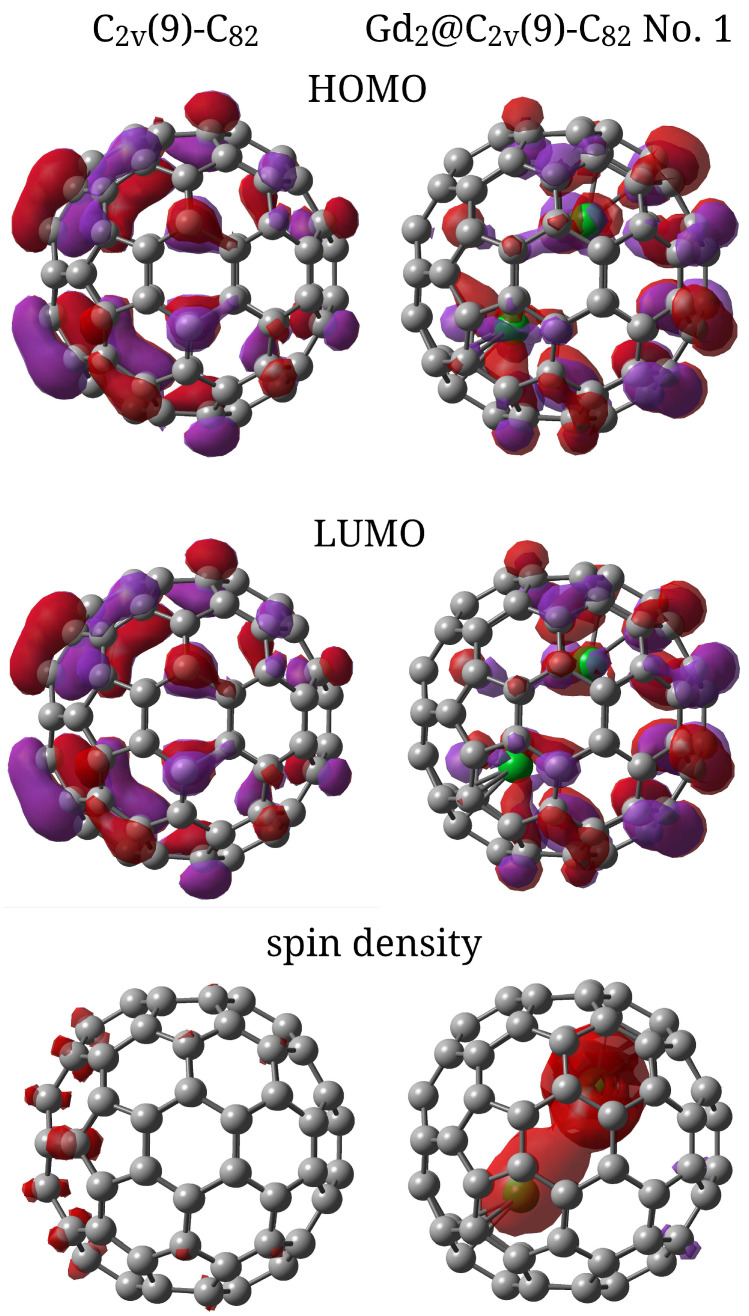
Isosurfaces of HOMO, LUMO, and spin density for C_2*v*_(9)-C_82_ and Gd_2_@C_2*v*_(9)-C_82_ No. 1.

The Hirshfeld charge on the C_82_ cage is similar for all isomers presented in Table 2 and is approximately −4.5 a. u., indicating a significant transfer of electron density to the carbon cage. Figure 3 shows the isosurfaces of HOMO and LUMO and the spin density for the Gd_2_@C_2*v*_(9)-C_82_ No. 1 EMF and the empty C_2*v*_(9)-C_82_. The calculated structures of HOMO and LUMO are quite similar both for the empty fullerene and the EMF. The greatest contribution to the structure of these orbitals in C_2*v*_(9)-C_82_ comes from the atoms near the six-membered ring through which the C_2_ symmetry axis passes. In the endohedral fullerene Gd_2_@C_2*v*_(9)-C_82_ No. 1, the Gd atoms do not make a significant contribution to the HOMO and LUMO structures. This indicates their stabilisation within the carbon cage and the dominant role of the cage in the chemical reactivity of endofullerene. The set of isosurfaces presented in Figure 3 confirms the conclusion concerning the transfer of valence electrons from metal atoms to the carbon framework. For an empty fullerene in the ground state, the spin density on individual atoms is small and does not exceed 0.1 a. u. In the case of endofullerene, a clear localisation of the bulk of the spin density on the gadolinium atoms is observed. This confirms that the magnetic properties of the complex are determined by the *f*-electrons of the encapsulated metal atoms. The red areas of the isosurfaces on the Gd atoms in Figure 3 visualise the preferential localisation of unpaired electrons.

Calculated IP values of Gd_2_@C_82_ are ∼25–35 eV lower than those for the corresponding C_82_. The EA values for EMF Gd_2_@C_82_ differ even more from the corresponding values for empty C_82_; the difference is ∼0.55–0.75 eV.

**Figure 4:**
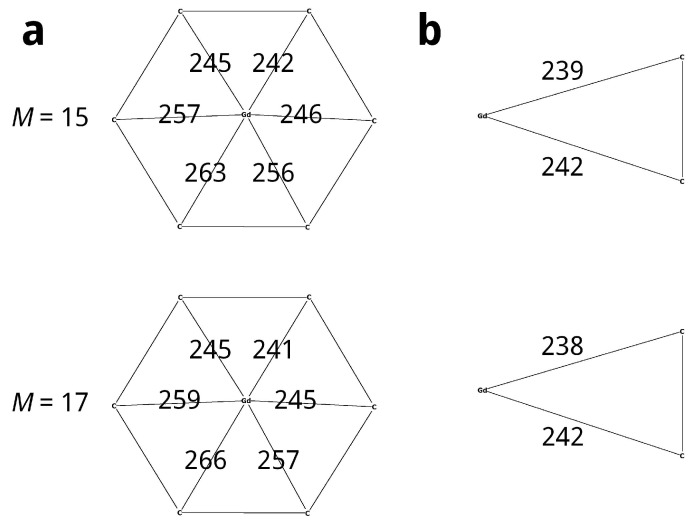
Optimised positions of the Gd atoms in Gd_2_@C_2*v*_(9)-C_82_ No. 1 relative to the nearest hexagonal ring (**a**) and to the nearest atoms of the 6–6 bond (**b**), pm.

The spin relaxation time and other magnetic properties of EMFs depend on their geometric structure and symmetry, which can be altered by transferring the structure to an excited state. In this regard, studying the excited state of Gd_2_@C_82_ with multiplicity 17 is of interest. Calculations show that the geometry of the most stable structure with *M* = 17 is very close to the geometry of the ground state with multiplicity 15 (Figure 4). The difference in the Go value between the ground and excited states is 12 kJ/mol. The C_82_ cage charge for the state with *M* = 17 is close to the corresponding value for the ground state (−4.527 a. u.) and amount to −4.513 a. u. The spin density on the carbon cage for the excited state is 1.181; i. e., there is a spin flip of a cage electron upon excitation of the EMF.

### 2.3. IR Spectra of Gd_2_@C_82_ EMF

The vibrational modes of Gd_2_@C_82_ can be categorised into three distinct groups:

- Endohedral (metal-related) Gd–Gd modes ν<200 cm^−1^, involving Gd–Gd stretching and vibrations of the metal cluster relative to the cage.
- Radial cage modes 200<ν<800 cm^−1^, dominated by out-of-plane atomic displacements that alter the cage’s curvature (including ‘squashing’ and ‘breathing’ motions).
- Tangential cage modes ν>800 cm^−1^, involving in-plane C–C stretching (graphen-like modes). Due to significant charge transfer from Gd_2_ to the cage, these modes exhibit strong IR intensities and noticeable redshift (toward lower frequencies) compared to empty C_82_

In the IR spectrum of Gd_2_@C_82_, the first six frequencies correspond to vibrations of the Gd atoms relative to the cage (Table 3). The noticeable differences in the IR spectra of the empty fullerene and the endohedral fullerene are caused by vibrations of the metal atom inside the carbon cage, whose shape is distorted due to the formation of metal–carbon bonds, charge transfer, and significant polarisation of the fullerene.

The $\nu_{5}$ frequencies of all Gd_2_@C_82_ isomers listed in Table 3 lie in the 137–149 cm^−1^ range which is close to the experimental Raman frequency, 151 cm^−1^ for Gd@C_82_ [38].

**Figure 5:**
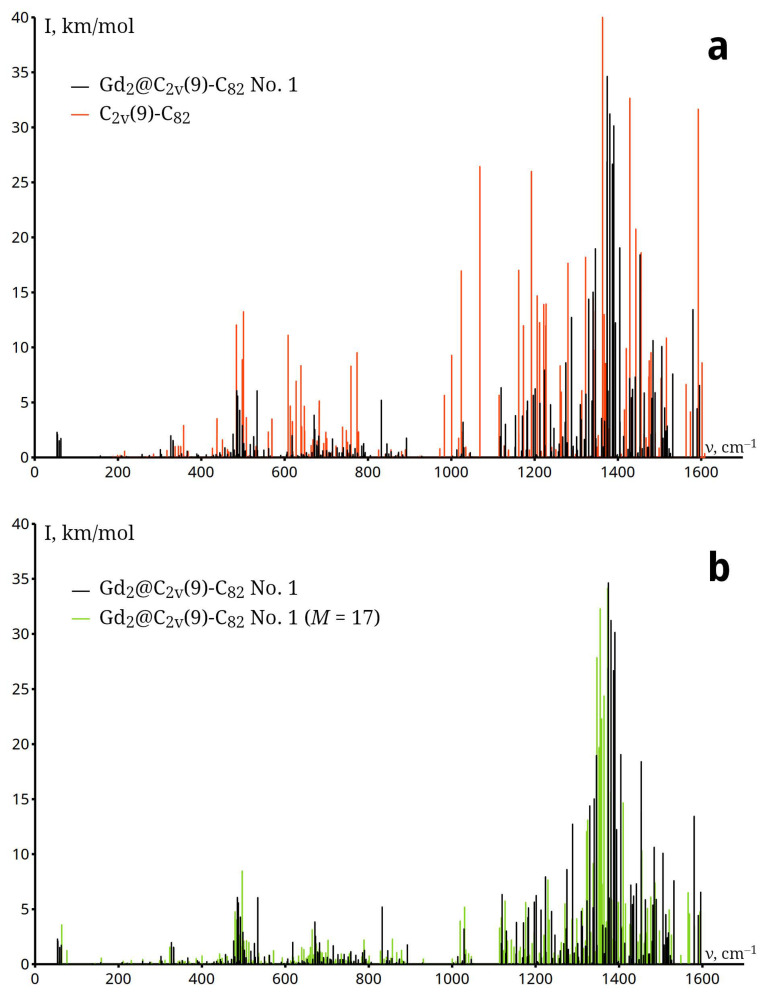
Comparison of IR spectra of Gd_2_@C_2*v*_(9)-C_82_ No. 1 and C_2*v*_(9)-C_82_ (**a**); Gd_2_@C_2*v*_(9)-C_82_ No. 1 and Gd_2_@C_2*v*_(9)-C_82_ No. 1, *M* = 17 (**b**).

During endofullerene formation, electrons are transferred from the metal atoms to the carbon cage. The increased electron density on the carbon cage weakens the bonds. This leads to a redshift of the cage’s vibrational modes compared to the neutral empty fullerene (observed in Figure 5 in the radial vibration line groups in the 300–500 cm^−1^ range and the stretching vibration lines in the 1100–1600 cm^−1^ range). Furthermore, charge transfer makes the molecule polar, which leads to the activation of lines in the IR spectrum of Gd_2_@C_82_ that were not visible in the spectrum of C_82_.

The low-frequency region of the vibrational spectrum (49–158 cm^−1^) is dominated by metal–cage (M–cage) $\nu_{1}$–$\nu_{6}$ modes, characterised by significantly high reduced masses (30–59 amu). These values, considerably exceeding the atomic mass of carbon, provide direct evidence of the collective participation of the heavy gadolinium atoms ($m_{\mathrm{Gd}}=157.25$ amu) in these vibrational motions.

Vibrational modes 1–4 correspond to the displacement of Gd atoms tangential to the fullerene interior surface, which corresponds to the lateral M–cage modes observed in monometallofullerenes. This group comprises two distinct types of motion:

- Shear vibrations of the Gd_2_ cluster, characterised by parallel displacement of the metal atoms along the axis perpendicular to the Gd–Gd axis.
- Torsional vibrations, involving antiparallel motion of the metal atoms within the cage.

Modes 5 and 6 represent displacements along the normal to the cage surface (longitudinal M–cage modes).

- Mode 5 (143.28 cm^−1^, μ=45.76 amu) is identified as a symmetric longitudinal mode (Gd–Gd stretching-like motion).
- Mode 6 (157.97 cm^−1^, μ=29.72 amu) corresponds to an antisymmetric longitudinal mode, described as a translational/confined motion of the Gd_2_ cluster.

Notably, mode 6 exhibits a lower reduced mass and a higher frequency, as compared to mode 5. This shift indicates a more pronounced coupling with the carbon framework during the antisymmetric displacement involving more carbon atoms. The steeper potential wall encountered by the dimer during this translation leads to enhanced vibrational frequencies, reflecting the strong confinement effects within the C_82_ cavity.

Structural optimisation of the Gd_2_@C_82_ isomers yields an interatomic Gd–Gd distance of at least 3.92 Å. This value significantly exceeds the bond length in bulk metallic Gd (3.64 Å), suggesting the absence of a formal covalent bond between the encapsulated metal atoms. Consequently, the dynamics of the Gd_2_ cluster are primarily governed by the balance between the Coulombic repulsion of the Gd^3+^ ions and their indirect interaction mediated by the π-system of the carbon cage. The observed vibrational behavior is consistent with the motion of two ions within a complex, multi-well potential energy surface defined by the host–guest coordination.

The spectra of the ground and excited states are very similar both in terms of the position and intensity of the lines, which is due to the similarity of the energy, geometry, and charge state of the Gd atoms in these states (Figure 5).

## 3. Methods

Endofullerenes encapsulating lanthanide metal atoms (especially gadolinium) are rather difficult systems to calculate, mainly due to problems with the convergence of the SCF (self-consistent field) procedure. Even for a single lanthanide atom, choosing the optimal calculation scheme is not an easy task [39]. We did not used any forced convergence commands. To reach convergence in the SCF procedure, the following approaches were used: (i) Changing the SCF procedure algorithm to one that is more computationally expensive and converges better. (ii) Adjusting the SCF procedure parameters. For example, using SCF=QC (Quadratic Convergence) in Gaussian or procedure=BFGS (Broyden–Fletcher–Goldfarb–Shanno) instead of the standard NR in Priroda package (see Table 4 below). (iii) Preliminary calculation of the cation (using a simpler scheme or the Hartree–Fock approximation). This calculation converges better; and the resulting wave function was used as an initial guess for the SCF procedure.

In this work, we test the four calculation schemes described in Table 4. To check the usability and accuracy of these calculation schemes, we performed a preliminary benchmark calculation of the ground and excited state geometry of Gd@C_2*v*_(9)-C_82_, as well as some structural and physical characteristics of the C_60_ fullerene and GdC_2_ particle in different schemes, which provide SCF convergence and acceptable computer resource requirements.

This research partly used computational resources of the HPC SEC shared research facilities at Volga Research and Education Center for Supercomputing Technologies, Lobachevsky State University in Nizhny Novgorod (HPC UNN). Molecular structures and molecular orbitals were visualised by GaussView^®^ version 3.09 [40] and ChemCraft version 1.8 [41] program packages. None of the calculated vibrational frequencies of the studied EMFs contain an imaginary part, which suggests that all optimised structures correspond to minimum points on the potential energy surface.

**Table 4:** Tested calculation schemes.

No.	Functional	Basis	Pseudopotential	Software Package
1	mPW3PBE [42]	def2-SVP [43]	def2-SVP [44]	Gaussian 09 [45]
2	B3PW91 [46]	def2-SVP [43]	def2-SVP [44]	Orca 3.0.4 [47]
3	PBE0 [48]	D95 [49]	SDD [50]	Orca 3.0.4 [47]
4	mPBE [51]	L1 * [52]	—	Priroda 23 [53]

* used with the spin-free Dirac–Coulomb Hamiltonian [54].

The following geometry and physical parameters were calculated for testing the calculation schemes:

$r_{5–6}$: length of the common edge of five- and six-membered rings in C_60_ fullerene;
$r_{6–6}$: length of the common edge of two six-membered rings in C_60_;
$\nu_{1}$–$\nu_{4}$:vibration frequencies known in the IR spectrum of C_60_ fullerene;
$E_{b}\left(C_{60}\right)$: binding energy per carbon atom in C_60_ fullerene;
$D^{\circ }\left({\mathrm{GdC}}_{2}\right)$: standard enthalpy of dissociation of the GdC_2_ molecule.

The experimental value of $E_{b}\left(C_{60}\right)$ was estimated as

$$E_{b}\left(C_{60}\right)=\Delta H_{f}^{\circ }\left(\mathrm{graphite}\right)-\frac{1}{60}\Delta H_{f}^{\circ }\left(C_{60}\right),$$

where $\Delta H_{f}^{\circ }\left(\mathrm{graphite}\right)=717$ kJ/mol is the standard enthalpy of formation of graphite [[32], pp. 5–9], and $\Delta H_{f}^{\circ }\left(C_{60}\right)=2530$ kJ/mol is the standard enthalpy of C_60_ formation [[55], Table 11].

The results of the calculations of the characteristics of C_60_ fullerene and the GdC_2_ particle are presented in Table 5. Our testing did not reveal a computational scheme that had an advantage in the accuracy of all the parameters simultaneously. For C_60_, scheme 3 gives the value of its binding energy to experiment. The enthalpy of dissociation of GdC_2_ is better reproduced in schemes 1 and 2. Scheme 4 gives the best agreement of the C_60_ vibration frequencies with respect to the experiment, and also has the highest performance.

**Table 5:** Calculated and experimental properties of C_60_ and GdC_2_ particles.

Property	Scheme 1	Scheme 2	Scheme 3	Scheme 4	Experiment
$r_{5–6}\left(C_{60}\right)$, pm	145.0	145.0	144.9	145.4	145.2 [56]
$r_{6–6}\left(C_{60}\right)$, pm	139.6	139.6	139.4	139.9	139.7 [56]
$\nu_{1}\left(C_{60}\right)$, cm^−1^	538	539	528	521	527 [57]
$\nu_{2}\left(C_{60}\right)$, cm^−1^	598	595	599	577	576 [57]
$\nu_{3}\left(C_{60}\right)$, cm^−1^	1243	1240	1241	1183	1182 [57]
$\nu_{4}\left(C_{60}\right)$, cm^−1^	1502	1498	1512	1434	1429 [57]
$E_{b}\left(C_{60}\right)$, kJ/mol	704	695	682	696	675
$D^{\circ }\left({\mathrm{GdC}}_{2}\right)$, kJ/mol	1256	1242	1212	1290	1255 ± 25 [58]

**Figure 6:**
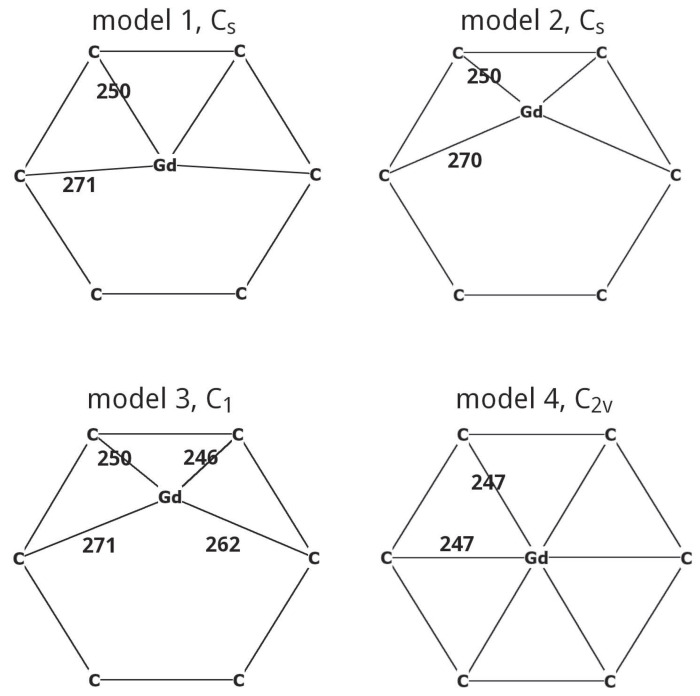
Optimised positions (in pm) of the Gd atom in Gd@C_2*v*_(9)-C_82_ relative to the nearest six-membered ring and Gd–C bond lengths; the experimental value: 249±3 pm [59].

Figure 6 shows the optimised positions of the Gd atom in Gd@C_2*v*_(9)-C_82_ relative to the nearest six-membered ring. Only scheme 4 predicts the position of the Gd atom on the C_2_ axis passing through the center of the ring, which is established experimentally. The difference in the electronic energy of the Gd@C_2*v*_(9)-C_82_ states with multiplicities M=9 and M=7 is 55, −17, −21, and 8 kJ/mol for schemes 1–4, respectively. Schemes 1 and 4 correctly reproduce the Gd@C_82_ ground state multiplet, but the 55 kJ/mol energy difference between the ground and excited states given by scheme 1 appears to be overestimated. These results suggest that only scheme 4 can correctly predict the geometry and multiplicity of the ground state of Gd@C_2*v*_(9)-C_82_, so it was chosen for further calculations.

## Figures and Tables

**Table 3:** Calculated vibrational frequencies (in cm^−1^) of the six most stable Gd_2_@C_82_ isomers related to Gd–cage vibrations.

Isomer	$\nu_{1}$	$\nu_{2}$	$\nu_{3}$	$\nu_{4}$	$\nu_{5}^{*}$	$\nu_{6}$
Gd_2_@C_2*v*_(9)-C_82_ No. 1	49	54	64	73	143	158
Gd_2_@C_2*v*_(9)-C_82_ No. 1 (M=17)	58	62	64	76	143	159
Gd_2_@C_2*v*_(9)-C_82_ No. 2	48	52	59	75	141	159
Gd_2_@C_2*v*_(9)-C_82_ No. 3	53	56	65	78	143	155
Gd_2_@C_*s*_(6)-C_82_ No. 1	62	72	76	83	149	157
Gd_2_@C_3*v*_(8)-C_82_ No. 1	54	55	59	63	137	157
Gd_2_@C_*s*_(6)-C_82_ No. 2	53	62	68	78	137	158

* Gd–Gd vibration.

## Data Availability

The data are available from the authors.
